# CONTINUOUS WORK-BASED LEARNING IN THE NURSING HOME SETTING

**DOI:** 10.1093/geroni/igac059.1517

**Published:** 2022-12-20

**Authors:** Judith Meijers

## Abstract

Nursing homes face challenges caused by increasing numbers of older adults with frailty and multimorbidity, and complexity of care in combination with decades of under resourcing and severe workforce challenges. In most countries the vast majority of direct care in LTC homes is provided by a large unregulated workforce that is largely unregulated, female, racialized and poorly remunerated. The results are severe difficulties in recruiting and retaining staff at all levels and a tendency to substitute less qualified workers for more highly qualified and more highly remunerated. Less well reported are the significant pressures that critical LTC home managers face. However, they are equally under duress especially in the wake of a global pandemic. Developing a culture of continuous learning and integrating this into daily nursing practice is one promising way to attenuate these challenges, ensure a high quality of care and a better equipped nursing staff. However, insight into strategies and interventions that contribute to such a continuous work-based learning culture in nursing homes is still relatively limited. In this symposium we will focus on different approaches to increasing workplace learning in long term care.

